# NLRR1 Is a Potential Therapeutic Target in Neuroblastoma and MYCN-Driven Malignant Cancers

**DOI:** 10.3389/fonc.2021.669667

**Published:** 2021-06-25

**Authors:** Atsushi Takatori, MD. Shamim Hossain, Atsushi Ogura, Jesmin Akter, Yohko Nakamura, Akira Nakagawara

**Affiliations:** Division of Innovative Cancer Therapeutics, Chiba Cancer Center Research Institute, Chiba, Japan

**Keywords:** neuronal leucine-rich repeat 1, neuroblastoma, epidermal growth factor receptor, monoclonal antibody, epitope mapping

## Abstract

Receptor tyrosine kinases (RTKs) receive different modulation before transmitting proliferative signals. We previously identified neuronal leucine-rich repeat 1 (NLRR1) as a positive regulator of EGF and IGF-1 signals in high-risk neuroblastoma cells. Here, we show that NLRR1 is up-regulated in various adult cancers and acts as a key regulator of tumor cell proliferation. In the extracellular domains of NLRR1, fibronectin type III (FNIII) domain is responsible for its function to promote cell proliferation. We generated monoclonal antibodies against the extracellular domains of NLRR1 (N1mAb) and screened the positive N1mAbs for growth inhibitory effect. The treatment of N1mAbs reduces tumor cell proliferation *in vitro* and *in vivo*, and sensitizes the cells to EGFR inhibitor, suggesting that NLRR1 is a novel regulatory molecule of RTK function. Importantly, epitope mapping analysis has revealed that N1mAbs with growth inhibitory effect recognize immunoglobulin-like and FNIII domains of NLRR1, which also indicates the importance of FNIII domain in the function of NLRR1. Thus, the present study provides a new insight into the development of a cancer therapy by targeting NLRR1 as a modulator of proliferative signals on cellular membrane of tumor cells.

## Introduction

Neuroblastoma (NB), originally arising from the sympathoadrenal lineage of the neural crest, is one of the most common extracranial solid tumors in childhood. NBs in patients less than 1 year of age often regress spontaneously, resulting in a favorable prognosis (1). In contrast, tumors found over 1 year of age are usually aggressive leading to poor prognosis. A subset of NB with poor prognosis is characterized by the presence of genetic aberrations, such as gain of chromosome 17q, loss of chromosome 11q, and amplification of *MYCN* oncogene (2, 3). MYCN is a nuclear transcription factor and one of the most important prognostic indicators of poor clinical outcome (4). In general, MYCN regulates cell proliferation through transcriptional regulation of its target genes in both positive and negative manners (5, 6). However, genes contributing to tumor growth and aggressiveness of NB under MYCN regulation still remain elusive.

Tumor growth is mediated by the activity of receptor tyrosine kinases (RTKs) functionally regulated by different mechanisms including gene expression, endocytosis, dephosphorylation, and crosstalk with other membrane proteins (7–9). Our previous studies have revealed that neuronal leucine-rich repeat 1 (NLRR1), a type І transmembrane protein, is associated with tumorigenesis by promoting cell proliferation through the activation of ERK mediated by EGF and IGF-1 (10) and negatively regulating anaplastic lymphoma kinase (ALK) (11) in NB, although the contribution of NLRR1 to other types of cancers is not understood. NLRR1 was originally identified in a cDNA project to seek new therapeutic target genes differentially expressed between favorable and unfavorable NBs (12, 13). The human NLRR family consists of three members, NLRR1, NLRR2, and NLRR3. NLRR1 expression is significantly high in advanced stages of NB with poor prognosis, whereas that of NLRR3 is significantly high in early stages of NB with good prognosis (14, 15). Interestingly, transcription of *NLRR1* and *NLRR3* is oppositely regulated by MYCN, a member of MYC family of oncogenes frequently amplified in aggressive NB. These previous findings suggested NLRR1 as an executer protein for aggressiveness of NB under MYCN regulation and a possible therapeutic target to control tumor growth.

In high-risk NB, despite a great improvement of its combinatorial therapy with surgical resection, intensive chemotherapy, radiotherapy, and immunotherapy, only 40 to 50% of patients survive long term (3, 16). Therefore, new and efficient therapeutic strategies are required to improve overall survival of the high-risk group. To date, clinical trials of molecular targeted therapy (e.g. EGFR, IGF-IR, or ALK) have been performed in pediatric solid tumors (17–19). However, more preclinical and clinical trials are needed to identify key targets that can be efficiently exploited therapeutically and help develop a patient-tailored therapy because NB is a heterogeneous tumor (3).

In the present study, we found the up-regulation of NLRR1 expression in various adult cancers and non-NB cell lines. Hybridomas producing monoclonal antibodies (mAbs) to extracellular part of NLRR1 were developed and subjected to screening assays. Monoclonal antibody against NLRR1 (N1mAb) with growth inhibitory effect was found to target the domains of NLRR1 responsible for its function to regulate cell proliferation. Furthermore, the treatment of N1mAb suppressed EGF signals, potentiated the effect of EGFR inhibitor, and decreased the tumor growth in mouse xenograft models.

## Materials and Methods

### Reagents and Antibodies

EGF and IGF-I were from Sigma (St. Louis, MO, USA). Complete protease inhibitor cocktail and phosphatase inhibitor cocktail were from Roche (Indianapolis, IN, USA); 5-bromo-2-deoxyuridine (BrdU) was from Sigma; 3,3′-Dithiobis(sulfosuccinimidylpropionate) (DTSSP) was from Thermo Fisher Scientific (Rockford, IL, USA). Antibodies against phospho-EGFR (#2236), phospho-ERK (#9101), phospho-Akt (#9271), EGFR (#4267), ERK (#9102), Akt (#9272), and myc tag (#2276) were from Cell Signaling Technology (Danvers, MA, USA); anti-*β*-III tubulin (Tuj1) antibody (#MMS-435P) was from Covance (Princeton, NJ, USA); anti-HA tag antibody (#11867423001) was from Roche; anti-BrdU antibody (#M0744) was from DakoCytomation (Glostrup, Denmark); anti-actin (#A5060) was from Sigma; and the sheep polyclonal anti-NLRR1 antibody (#AF4990) was from R&D Systems (Minneapolis, MN, USA). AG1478 was from Calbiochem (Darmstadt, Germany). Lung and prostate tissue lysate arrays (Tissue Lysate Dipstick Array) were from Protein Biotechnologies (Ramona, CA, USA).

### Quantitative Real-Time PCR

Total RNA was extracted from 12 lung adenocarcinoma or nine squamous cell carcinoma and adjacent non-cancerous tissues as well as NB and non-NB cell lines using TRIzol reagent (Invitrogen) according to the manufacturer’s instructions, and reverse transcription was performed with SuperScript II reverse transcriptase (Invitrogen). qRT-PCR was carried out using 7500 Real-Time PCR System (Applied Biosystems), according to the manufacturer’s protocol. TaqMan probe for *NLRR1* (Assay ID: Hs00979743_m1) and *β*-*actin* control reagent kit were purchased from Applied Biosystems. The mRNA levels of each gene were standardized by *β*-*actin*. All human neuroblastoma specimens used in the present study were obtained at various institutions and hospitals in Japan and provided to the Chiba Cancer Center Neuroblastoma Tissue Bank with appropriate informed consent. The procedure of this study was reviewed and approved by the internal review board of Chiba Cancer Center.

### Cell Culture

HEK293 and MCF7 cells were cultured in Dulbecco’s Modified Eagle Medium (DMEM; Sigma) supplemented with 10% fetal bovine serum (FBS; Invitrogen, Carlsbad, CA, USA). Human neuroblastoma SH-SY5Y and SK-N-BE(2) cells were maintained in RPMI 1640 (Sigma) supplemented with 10% FBS. HEK293 cells were obtained from the JCRB Cell Bank and MCF7 and SH-SY5Y cells were from ATCC, while SK-N-BE(2) cells were purchased from the European Collection of Authenticated Cell Cultures. Mycoplasma contamination was tested by Mycoplasma Detection Set (Takara), and short tandem repeat analysis was performed for cell authentication (Promega). Transient transfection with C-terminal hemagglutinin (HA) or myc-tagged human NLRR1 plasmids was performed using Fugene HD (Roche) or Lipofectamine2000 (Invitrogen) according to the manufacturer’s instructions. Seventy to eighty percent confluent monolayers of transfected cells were treated with growth factors for the indicated times after 16 h serum starvation, and cell lysates were subjected to western blot analyses. Cell proliferation was determined using a Cell Counting Kit-8 (Dojindo, Japan).

### Generation of NLRR1 Stable Knockdown Cell Lines Using shRNA Lentiviruses

SK-N-BE cells were infected with four different MISSION lentiviral particles encoding NLRR1 shRNAs (Sigma). NLRR1 shRNA target sequences were as follows: #1, CCGGCCACAACTTTGCGTATGTGAACTCGAGTTCACATACGCAAAGTTGTGGTTTTTTG; #2, CCGGCCACCTGAACTCCAACAAATTCTCGAGAATTTGTTGGAGTTCAGGTGGTTTTTTG; #3, CCGGGCTGAACAACAATGCCTTGAACTCGAGTTCAAGGCATTGTTGTTCAGCTTTTTTG; and #4, CCGGGCTAGACTTGTTACCTTCGTTCTCGAGAACGAAGGTAACAAGTCTAGCTTTTTTG. Stable cell lines were generated by selection with puromycin (0.8 µg/ml). To exclude the possibility of off-target effects of NLRR1 shRNAs, 3′UTR-targeted #4 shRNA stably expressing SK-N-BE cells were transiently introduced with pcDNA3-NLRR1-HA expression plasmid and subjected to cell proliferation assays, as above.

### Histology and Immunostaining

Human tissue array slides were obtained from Super Bio Chips (Seoul, Korea) and subjected to immunostaining using polyclonal anti-NLRR1 antibody (1:25). BrdU was injected intraperitoneally into mice bearing tumors at a dose of 100 μg/g body weight 24 h before autopsy. Tumor tissues were fixed in 4% paraformaldehyde, dehydrated with a graded ethanol series, and embedded in paraffin. Sections (4 µm) were deparaffinized by immersion in xylene and rehydrated, followed by immunostaining for BrdU. Number of BrdU-positive cells was counted in four high power fields (hpfs) of each tumor at ×400 magnification.

### Immunoprecipitation and Western Blot Analyses

HEK293 cells expressing HA-tagged and myc-tagged NLRR1 were treated with cross-linker (DTSSP) for 2 h at 4°C. The cell lysates (500–750 μg) were incubated with appropriate antibodies for 1 h at 4°C, followed by incubation with protein G-agarose at 4°C overnight. After extensive washing of the beads with lysis buffer, the immunoprecipitates were detected by western blot analyses. Total cell lysates were resolved by SDS-PAGE followed by western blot detection using the indicated antibodies (1:1,000).

### Biotin Labeling on Cell Surface

HEK293 cells were transiently transfected with C-terminal tagged human NLRR1, NLRR2, or NLRR3 plasmids and treated with EZ-Link Sulfo-NHS-LC-LC-Biotin (Thermo Fisher Scientific) or vehicle alone for 30 min at room temperature. Cell lysates (500 μg) were incubated with NeutrAvidin Agarose Resin (Thermo Fisher Scientific). The precipitated proteins were resolved by SDS-PAGE followed by western blot detection using appropriate antibodies.

### Generation of Anti-Human NLRR1 Monoclonal Antibodies

The monoclonal antibodies were generated against extracellular domain of NLRR1 (MBL, Nagoya, Japan). The purified proteins of NLRR1 extracellular domain and complete Freund’s adjuvant (1:1) were injected into mice. Three days after the final injection, lymph-node cells were removed from immunized mice and were fused with P3U1 myeloma cells at a ratio of 5:1 by the polyethyleneglycol-400 procedure. Cultured supernatants of the hybridomas were screened in transfectants expressing NLRR1 by flow cytometric analysis (FCM) and the binding assay in 96-well plate coated with NLRR1 proteins. After cloning of the hybridomas which showed the positive results in FCM and/or the binding assay, the culture supernatants from wells were tested for growth inhibitory effect by culturing CHP134 cells at 1 × 10^5^/ml in the medium containing 50% of the conditioned medium from the hybridomas. For the positive hybridomas, the monoclonal antibodies were purified by protein A Sepharose column chromatography by MBL.

### Characterization of NLRR1 mAb

The isotype of each of the N1mAbs was determined by MBL. The plasmids for NLRR1 lacking the extracellular domains (ΔLRR, ΔIg, and ΔFNIII) were prepared using Infusion (Clontech, Mountain View, CA, USA). Peptide microarray was generated by JPT Peptide Technologies GmbH (Berlin, Germany) and analyzed by SureScan Microarray Scanner (Agilent Technology, Santa Clara, CA, USA). For flow cytometry, HEK293 cells were transiently transfected with the plasmids of NLRR1 lacking the extracellular domains and resuspended in phosphate-buffered saline (PBS). The cells were incubated with N1mAbs for 1 h at 4°C followed by the incubation with Alexa Fluor 488-labeled anti-mouse IgG (Thermo Fisher Scientific). The cells were analyzed using FACSCalibur (BD, San Diego, CA, USA).

### Tumor Growth Inhibition Study

SH-SY5Y cells stably expressing NLRR1 were established by transfection followed by selection with G418 at concentration of 600 µg/ml for about 4–6 weeks (10). Seven-week-old SCID mice (Charles River Laboratories) were subcutaneously inoculated with 5 x 10^6^ SH-SY5Y cells stably expressing NLRR1 and CHP134 cells in 0.1 ml of PBS/Matrigel. After implantation, tumor sizes were measured using the following formula: [(width)^2^ × length]/2. After tumors became >75 mm^3^, mice were randomized into two groups and intraperitoneally administered with vehicle or NLRR1 monoclonal antibody #281 twice a week for 3 weeks. For *in vivo* imaging, N1mAb 281 was labeled with HiLyte Fluor 750 using AnaTag Protein Labeling Kit (AnaSpec, Fremont, CA, USA) and visualized using a Lumazone imaging system (Roper Scientific, Tucson, AZ, USA) after injection into the tail vein. All mice were maintained in a specific pathogen-free animal facility. All animal experiments were approved by the Animal Care and Use Committee of Chiba Cancer Center Research Institute.

### Statistical Analysis

Student’s *t*-tests (two-tailed) and ANOVA tests were employed to examine the differences between two groups and that of differences between more than two groups, respectively. In xenograft study, the difference between groups was evaluated by two-way repeated measures ANOVA followed by Bonferroni posttest. *, *P* < 0.05; **, *P* < 0.01; and ***, *P* < 0.001 *versus* saline at each time; ^##^, *P* < 0.01 *versus* saline group.

## Results

### NLRR1 Expression Is Up-Regulated in Many Cancers

**Figure 1:**
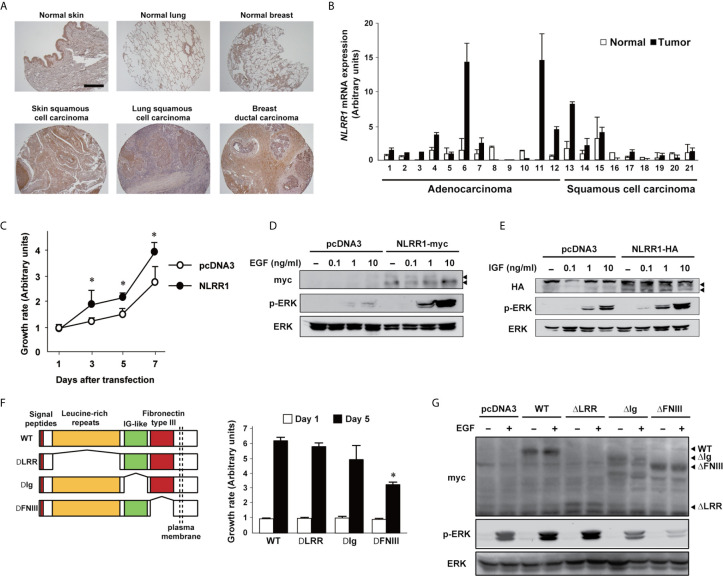
NLRR1 is up-regulated in various cancers and the Ig and FNIII domains are responsible for its function. **(A)** Immunohistochemistry using anti-N-terminal NLRR1 antibody in human normal and cancerous skin, lung, and breast tissue. The scale bar represents 500 μm. **(B)** Relative expression levels of NLRR1 mRNA in normal and lung carcinoma tissues (twelve adenocarcinoma and nine squamous cell carcinoma). Relative expression levels of *NLRR1* mRNA were determined by calculating the ratio between *β-actin* and *NLRR1*. **(C)** Overexpression of NLRR1 tagged with myc in NLRR1 low-expressing MCF7 cells promotes cell proliferation. Quantification of cell proliferation was performed by WST-8 assays. Results are given as mean ± SD. *P < 0.05, compared to pcDNA3. **(D, E)** NLRR1-expressing cells show ERK activation upon EGF and IGF treatment at low concentrations. MCF7 cells were transfected with pcDNA3-NLRR1 and treated with different concentrations of EGF **(D)** or IGF **(E)**. Ten minutes after treatment, the cell lysates were collected. Arrowheads, glycosylated NLRR1. **(F)** Deletion of FNIII domain resulted in a marked suppression of cell growth in MCF7 cells. Wild-type (WT) of NLRR1 or deletion mutants lacking leucine-rich repeats (LRR), immunoglobulin-like (Ig) or fibronectin type III (FNIII) were transiently expressed in MCF7 cells and subjected to WST-8 assays. Data were normalized to the results at Day 1 and indicated as mean ± SD. *P < 0.05, compared to WT. **(G)** FNIII domain is responsible for enhancing ERK phosphorylation upon EGF treatment. MCF7 cells expressing NLRR1 deletion mutants were treated with EGF (10 ng/ml) for 10 min, and the cell lysates were subjected to western blot analyses.

NLRR1 expression is significantly high in advanced stages of NBs with poor clinical outcome (20). High expression of *NLRR1* was also detected in NB cell lines and non-NB cell lines (**Figure S1A**). Here, we further examined NLRR1 expression in various cancer tissues. Immunohistochemistry revealed strong staining in cancer tissues from skin, lung, and breast as compared with the corresponding normal tissues (**Figures 1A** and **S1B**). Higher expression of NLRR1 in adult cancer tissues (lung and prostate) compared to normal tissue was also indicated by tissue lysate arrays (**Figure S1C**). To confirm the elevated expression of *NLRR1*, we measured mRNA expression in primary lung cancers by quantitative real-time RT-PCR (qRT-PCR). High expression of *NLRR1* was observed in tumorous tissues; eight out of twelve adenocarcinomas and three out of nine squamous cell carcinomas showed more than two-fold higher expression of *NLRR1* than normal tissues (**Figure 1B**). These data suggest that NLRR1 may contribute to the malignant status and serve as a biomarker not only in NB, but also in adult cancers.

### NLRR1 Increases Cell Proliferation by Enhancing the Cellular Signals of EGF and IGF

Similar to our previous observation in NB cells (10), overexpression of NLRR1 increased the cell growth in NLRR1-low-expressing MCF7 breast cancer cells (**Figure 1C**) and the activation of ERK in the cells was enhanced when treated with EGF and IGF in dose-dependent manner (**Figures 1D, E**) and time-dependent manner (**Figure S2**). To confirm the requirement of NLRR1 for cell proliferation, we performed NLRR1 depletion using lentiviral shRNAs in SK-N-BE NB cells with a moderate level of NLRR1 expression (10). Four different lentiviruses were prepared for stable cell lines and NLRR1 knockdown dramatically diminished cell proliferation (**Figure S3A**). The reduced proliferation with shRNA targeting 3′-UTR region of *NLRR1* was recovered by exogenous NLRR1 expression in a dose-dependent manner (**Figure S3B**), suggesting that NLRR1 expression is crucial to maintain normal cell proliferation.

### FNIII Domain Is the Functional Region of NLRR1 Required for Enhancing Growth Signaling

NLRR1 is a glycosylated transmembrane protein with external leucine-rich repeats (LRRs), immunoglobulin-like (Ig), fibronectin type III (FNIII) domains and a short intracellular tail. To identify the functional domains of NLRR1, we constructed the expression vectors of NLRR1 with the deletions in extracellular domains (ΔLRR, ΔIg, and ΔFNIII). Cell growth assay in MCF7 cells expressing the deletion mutant NLRR1 revealed that the deletion of FNIII domain significantly reduced the cell growth compared to wild-type NLRR1 (**Figure 1F**). In addition, the phosphorylation of ERK upon EGF treatment was diminished in ΔFNIII-expressing cells, while NLRR1 lacking ΔLRR had a comparable phospho-ERK. Of note, the deletion of Ig domain resulted in the reduced activation of ERK upon EGF treatment (**Figure 1G**). These data suggest that FNIII domain is a responsible domain of NLRR1 to enhance EGF signaling and increase cell proliferation and that Ig domain has an auxiliary function to support FNIII domain.

### N1mAb Suppresses Cell Proliferation

**Figure 2:**
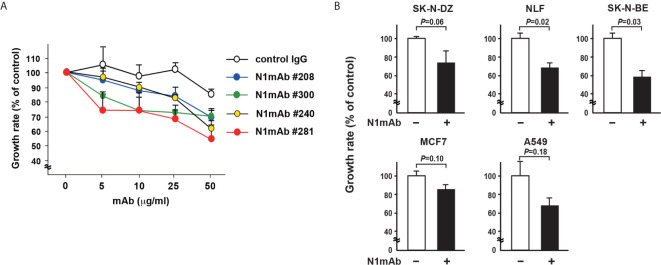
Generation of monoclonal antibodies against the extracellular domain of NLRR1 with growth inhibitory effect. **(A)** CHP134 NB cells were cultured in the medium containing varying amounts of the purified candidate mAbs. Four days after treatment, the proliferation of cells was subjected to WST-8 assays. Data were normalized to the results for untreated cells and represented as percentage of control (mean ± SD). **(B)** NB cells (SK-N-DZ, NLF, SK-N-BE), breast cancer cells (MCF7), and lung cancer cells (A549) were treated with N1mAb 281 at 100 μg/ml for 5 days. Quantification of cell proliferation was performed by WST-8 assays. Data are presented as the mean ± SD. The *P*-value was determined by the unpaired *t*-test.

To produce mAbs against NLRR1, the purified proteins of NLRR1 extracellular domain were used to immunize mice. To obtain a mAb that blocks the function of NLRR1, we tested cultured supernatants from candidate hybridomas sequentially by FCM and ELISA (**Figure S4A**). The positive cultured supernatants were further examined in growth inhibition assays using NLRR1-expressing cells. The cell growth treated with NLRR1 IgG mAbs of No. 240, 281, and 300, was suppressed, while little effect was observed by FCM-negative NLRR1 mAb No. 23 (**Figure S4B**). After purification of the positive IgG mAbs, CHP134 NB cells were cultured in the medium containing varying amounts of the purified candidate mAbs. Four days after treatment, the proliferation of cells was greatly inhibited by NLRR1 mAbs, especially by N1mAb 281 and 300, in a dose-dependent manner (**Figure 2A**). Among the mAbs, N1mAb281 was more effective in suppressing cell proliferation compared to other mAbs, and it was selected for further investigation. The growth inhibition assay using N1mAb281 was performed in NB cells (SK-N-DZ, NLF, SK-N-BE), breast cancer cells (MCF7), and lung cancer cells (A549). The treatment with high dose of N1mAb281 (100 μg/ml) significantly suppressed cell growth in NLF and SK-N-BE cells, while the marginal inhibitory effect was observed in SK-N-DZ, MCF7 and A549 cells (**Figure 2B**).

### The Treatment of N1mAb Inhibits Tumor Growth

**Figure 3:**
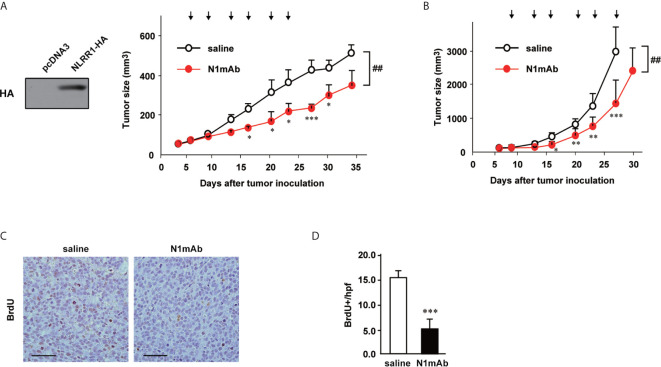
NLRR1 monoclonal antibody inhibits NB tumor growth *in vivo*. **(A)** Female SCID mice were subcutaneously inoculated with SH-SY5Y cells stably expressing NLRR1 and randomized into two groups (seven mice per group). Mice were i.p. injected with saline or N1mAb 281 (100 µg) twice a week for 3 weeks (arrows). Data are shown as mean tumor volume ± SD. **(B)** SCID mice (seven mice per group) bearing CHP134 xenografts were injected i.p. with saline or N1mAb 281 (200 µg) twice a week for 3 weeks (arrows). The difference between groups was evaluated by two-way repeated measures ANOVA followed by Bonferroni posttest. *P < 0.05; **P < 0.01; and ***P < 0.001 *versus* saline at each time; ^##^ *P* < 0.01 *versus* saline group. **(C)** Twenty-four hours after BrdU administration (100 µg/g of body weight), all mice bearing CHP134 tumors in **(B)** were sacrificed and the paraffin sections of tumor tissues were subjected to BrdU immunohistochemistry to quantify cell proliferation. **(D)** The number of BrdU-positive cells in tumor tissues was counted per high power field (hpf). Data are mean ± SD obtained from seven mice for each group.

We next checked the efficacy of N1mAb treatment in xenograft model using SCID mice bearing NLRR1-stably expressing SH-SY5Y (SH-SY5Y/NLRR1) tumors. Even at relatively low dose (100 µg, twice a week), treatment of N1mAb led to significant suppression of tumor growth of SH-SY5Y/NLRR1 cells, strongly supporting the growth inhibitory effect of N1mAb (**Figure 3A**). The localization of N1mAb in the tumor was confirmed using near-infrared fluorescent-labeled N1mAb 281 systemically administered by tail vein injection (**Figure S5**). We further determined the antitumor effect of N1mAb on endogenously expressed NLRR1 in CHP134 tumor xenograft. As shown in **Figure 3B**, N1mAb treatment (200 µg, twice a week) significantly reduced the tumor growth with 51% tumor regression without a loss of body weight (data not shown). To examine the effect of N1mAb in cell proliferation *in vivo*, we performed immunohistochemistry for BrdU-labeled cells and found that the number of BrdU positive cells was significantly decreased in N1mAb-treated CHP134 xenograft tumors (**Figures 3C, D**). Hence, we concluded that the treatment of N1mAb induces growth inhibitory effect *in vitro* and *in vivo*.

### N1mAb 281 Binds to Ig and FNIII Domains of NLRR1

**Figure 4:**
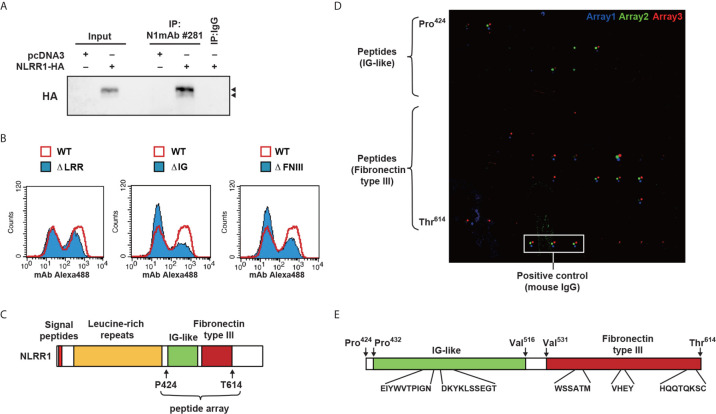
Epitope mapping of N1mAb 281 using a peptide microarray. **(A)** HA-tagged NLRR1 was expressed in HEK293 cells, and the cell lysates were subjected to immunoprecipitation using N1mAb 281. Normal mouse IgG was used as a negative control. NLRR1 protein was detected by anti-HA antibody. Input represents 2% of the total lysates used for immunoprecipitation. Arrowheads, glycosylated NLRR1. **(B)** HEK293 cells were transfected with plasmids encoding wild-type (WT) of NLRR1 or deletion mutants lacking leucine-rich repeats (LRRs), immunoglobulin-like (Ig), or fibronectin type III (FNIII). The binding of N1mAb 281 was assessed by flow cytometry to find the domains recognized by the antibody. **(C)** Schematic representation of NLRR1. Peptide microarray was prepared for Ig and FNIII domains by spotting 91 overlapping 12-mer peptides and immunostained with N1mAb 281. **(D)** Bound antibodies were detected with Alexa 546-labeled anti-mouse IgG. The scanned arrays obtained from three independent experiments were indicated. **(E)** Sequence of the peptides positive for all three independent experiments are indicated.

To elucidate the mechanism of growth inhibition by N1mAb, the binding property of N1mAb 281 was examined by immunoprecipitation and indirect flow cytometry. HEK293 cells overexpressing HA-tagged NLRR1 were subjected to immunoprecipitation assay. As shown in **Figure 4A**, HA-tagged NLRR1 was immunoprecipitated with N1mAb 281. To further examine a responsible region of NLRR1 protein for the binding of N1mAb 281, HEK293 cells transfected with expression vectors of NLRR1 wild-type, ΔLRR, ΔIg, or ΔFNIII were immunostained with N1mAb 281 followed by Alexa Fluor 488 conjugated anti-mouse IgG. The population of NLRR1-expressed cells detected by N1mAb 281 was decreased by deletion of Ig and FNIII domains, while NLRR1 lacking LRR domain exhibited little change compared to NLRR1 wild-type (**Figure 4B**). According to these results, we postulated that N1mAb 281 binds to Ig and/or FNIII domains of NLRR1. To identify the epitope sites recognized by N1mAb 281, we next generated 12-amino acid peptide microarray against Ig and FNIII domains from Pro 424 to Thr 614 (**Figure 4C**). As shown in **Figure 4D**, positive signals on microarrays in three independent experiments of immunostaining with N1mAb 281 were obtained mainly with the two regions of spots in Ig domain and three regions in FNIII domain (**Figure 4E**). These data suggest that the N1mAb 281 can recognize the discontinuous epitopes of Ig and FNIII domains in the extracellular part of NLRR1. We failed to detect the bands of NLRR1 by western blot analysis using N1mAb 281 (data not shown), indicating that the antibody binds to the two domains of NLRR1 in a conformation-dependent manner. We further examined the epitope sites recognized by the other N1mAbs, 300 and 240 (growth inhibitory effect positive and negative, respectively). N1mAb 300, which showed a comparable growth inhibitory effect to N1mAb 281 (**Figure 2A**), detected the peptides from both Ig and FNIII domains, whereas N1mAb 240 with a weak growth inhibitory effect showed no signals in the peptide microarray (**Figure S6**). These results suggest that the growth inhibitory effect of N1mAb 281 is exerted through binding to Ig and FNIII domains of NLRR1.

### Combinatory Use of N1mAb With EGFR Inhibitor Is Effective in the Resistant Cancer Cells

**Figure 5:**
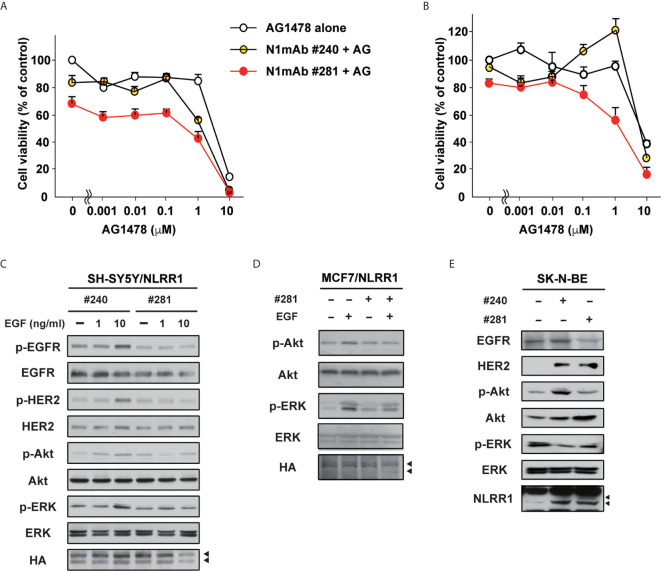
N1mAbs potentiates EGFR inhibitor-induced growth suppression. **(A)** NLRR1-stably expressing SH-SY5Y cells were treated with N1mAb 281 (25 μg/ml) and different concentrations of AG1478 (AG) for 72 h Data are represented as mean ± SD. Quantification of cell proliferation was performed by WST-8 assays. Data were normalized to the results for untreated cells and represented as percentage of control (mean ± SD). **(B)** AG1478-resistant A549 cells were treated with N1mAb 281 and different concentrations of AG1478 for 72 h **(C)** NLRR1-stably expressing SH-SY5Y cells were starved and treated with N1mAb 240 or 281 at 25 μg/ml for 3 h, followed by EGF treatment at the indicated concentration for 10 min. Cell lysates were subjected to western blot analyses. Arrowheads, glycosylated NLRR1. **(D)** MCF7 cells overexpressing NLRR1 were starved and treated with N1mAb 281 at 25 μg/ml for 3 h, followed by EGF treatment (1 ng/ml) for 10 min. The cell lysates were subjected to western blot analyses. **(E)** To check the effect of the long-term treatment of N1mAbs, SK-N-BE cells were incubated with N1mAbs (30 μg/ml) for 7 days with medium change every 2 days, and the cell lysates were subjected to western blot analyses.

Next, we tested N1mAbs for the combinatorial effect with EGFR inhibitor because previous reports showed that EGFR is expressed in NB and adult cancers and could be a therapeutic target for these tumors (21, 22). The results in **Figure 5A** demonstrate that the additional treatment with N1mAb 281 resulted in the reduced cell proliferation at low concentration of AG1478 EGFR inhibitor. Compared with AG1478 alone, treatment with N1mAb 281 reduced 40% of viable cells co-treated with AG1478. In AG1478-resistant lung cancer A549 cells, N1mAb #281 treatment increased the sensitivity to AG1478 treatment (**Figure 5B**). A similar result was obtained by N1mAb 300 (**Figure S7**). In addition, the activation of EGF signals was examined in N1mAb pre-treated SH-SY5Y/NLRR1 cells. The phosphorylation of EGFR, HER2, and the downstream molecules, Akt and ERK, was greatly decreased by N1mAb 281 treatment (**Figure 5C**). The reduced downstream signals upon EGF treatment were also observed in NLRR1-stably expressing MCF7 cells (**Figure 5D**). Because NLRR1 knockdown in SK-N-BE cells resulted in the impaired cell proliferation (**Figure S3**), we treated the cells with N1mAb for 7 days to check the influence on EGF signaling molecules. As shown in **Figure 5E**, N1mAb treatment down-regulated ERK activation, although the reduced expression of EGFR and phosphorylation of Akt was observed only in N1mAb 281-treated cells. Of note, N1mAb treatment increased the expression of HER2 and NLRR1, implying a mechanism of feedback up-regulation of the expression against NLRR1 inhibition. Thus, N1mAb treatment suppresses EGF signals and sensitizes the cells to the growth suppression induced by EGFR inhibitor treatment.

**Figure 6:**
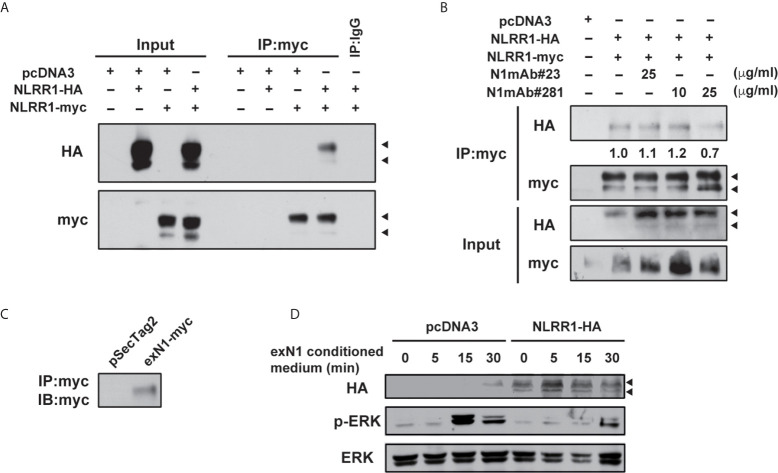
N1mAb 281 blocks the self-interaction of NLRR1 on cell surface. **(A)** HA-tagged and myc-tagged NLRR1 were expressed in HEK293 cells and the cell lysates were collected after crosslinking with membrane-impermeable DTSSP. Immunoprecipitation was performed using anti-myc antibody, and the immunoprecipitates were subjected to western blot analyses. Arrowheads, glycosylated NLRR1. **(B)** HA-tagged NLRR1 co-immunoprecipitated with myc-tagged NLRR1 was reduced by N1mAb 281 treatment. HEK293 cells expressing HA-tagged and myc-tagged NLRR1 were treated with N1mAb 23 or 281 at the indicated concentration for 3 h The cell lysates were collected after DTSSP treatment and subjected to immunoprecipitation using anti-myc antibody. **(C)** HEK293 cells were transfected with pSecTag2 vector (Invitrogen) containing the extracellular domain of NLRR1 tagged with myc (exN1). The secreted exN1 protein in the conditioned medium supplemented with 10% FBS was confirmed by immunoprecipitation using anti-myc antibody. **(D)** MCF7 cells were transfected with empty or pcDNA3-NLRR1-HA vectors and treated with the conditioned medium containing exN1 **(C)** for the indicated time. The cell lysates were collected and subjected to western blot analyses.

To understand the mechanism how N1mAb inhibits the function of NLRR1, we first performed immunoprecipitation assay using vectors for two different tagged NLRR1 and found that NLRR1 proteins form self-multimers (**Figures 6A** and **S8A**). Because cell surface biotinylation confirmed the localization of NLRR1 to the cell surface (**Figure S8B**), we postulated that NLRR1 might be present on the cell surface as self-multimer. Therefore, we pre-treated the cells expressing the two different tagged NLRR1 with N1mAbs and found that N1mAb 281 blocked the self-multimerization of NLRR1, whereas N1mAb 23, a negative control antibody, showed little or no effect (**Figures 6B** and **S8C**). To further examine the importance of NLRR1 self-interaction on its function, we prepared conditioned medium from HEK293 cells expressing extracellular domain of NLRR1 (**Figure 6C**). Treatment of the conditioned medium containing 10% FBS induced phosphorylation of ERK in control MCF7 cells, whereas NLRR1-expressing cells had no phosphorylation of ERK at 15 min after treatment (**Figure 6D**). These data suggest that the inhibition of self-multimerization by the antibody or free extracellular protein of NLRR1 blocks the function of NLRR1 and represses growth-promoting intracellular signals.

## Discussion

We demonstrate that NLRR1 expression is up-regulated in adult cancer tissues including lung and breast in addition to unfavorable NBs. NLRR1 is involved in determining the malignant status of cancer cells by enhancing the proliferative signaling of EGFR and IGF-IR. This is unique to NLRR1 among NLRR family members despite their close similarity in protein structures with high evolutional conservation (15). EGFR family receptors and IGF-IR, expressed in a wide variety of human cancers including NB, contribute to cell proliferation and tumor progression (23, 24). However, their expression levels in NB show no apparent correlation with the tumor stages (21, 25). On the other hand, high levels of NLRR1 expression are significantly associated with poor prognosis of NB (14, 20). Therefore, it is likely that the clinical significance of EGFR and IGF-IR in the pathogenesis of NB is at least in part inferred from their co-expression with NLRR1.

Transmembrane proteins that function to regulate cell proliferation have been the most popular candidates for therapeutic targets to develop a novel remedy against cancers. In particular, kinase inhibitors and neutralizing antibodies have been developed against RTKs of which the knockdown expression shows a great repression of cell growth and survival. Given that NLRR1 knockdown repressed NB cell proliferation, these lines of evidence regarding NLRR1 function in the present study may provide us an attractive scientific basis for targeting NLRR1. Three possible mechanisms of the inhibitory effect by single usage of NLRR1 antibody are hypothesized: [1] inhibition of NLRR1 self-dimerization which is shown in **Figure 6B**, although it is not clear yet whether N1mAb can recognize the dimeric conformation of NLRR1 Ig and FNIII domains, [2] induction of NLRR1 internalization, and [3] competitive inhibition of an endogenous unknown ligand for NLRR1. Further experiments may answer these remaining questions partly by investigating NLRR1 trafficking upon antibody treatment.

Our present characterization of epitopes recognized by N1mAbs revealed that N1mAbs with growth inhibitory activity bind to Ig and FNIII domains of NLRR1 (**Figure 4**). It is noteworthy to mention that, by the deletion mutant experiments, the same domains of NLRR1 were demonstrated to be required for its function in the regulation of cell proliferation (**Figure 1**). These data suggest that the Ig and FNIII domains are ideal antigen to raise a therapeutic antibody with potent growth inhibitory activity for malignant tumors with NLRR1 expression. The epitope sites of human NLRR1 recognized by N1mAbs have 100% identity to the sequence corresponding to mouse Nlrr1. The treatment of N1mAb in mice showed no influence on the gain of body weight and health condition, suggesting that the possible adverse effects by anti-NLRR1 therapy might be small.

We also tested combinatorial use of N1mAb with EGFR kinase inhibitor. At present, it has been proposed that partial inhibition of multiple targets could be more effective than full inhibition of a single molecule in NB therapy (3, 26). Indeed, N1mAb has more intensive inhibitory effect in combination with EGFR inhibitor, which may offer a novel therapeutic option to address some issues including acquired resistance and adverse effects arising from molecular targeting treatments (27). Thus, we propose here that NLRR1 is a novel molecular target for treating particular cancers including NB and that its function to regulate growth signals is dependent on its extracellular domain which can be a target for antibody-based therapy of NLRR1-expressing cancers.

## Data Availability Statement

The raw data supporting the conclusions of this article will be made available by the authors, without undue reservation.

## Ethical Statement

Ethical review and approval was not required for the study on human participants in accordance with the local legislation and institutional requirements. Written informed consent for participation was not required for this study in accordance with the national legislation and the institutional requirements. The animal study was reviewed and approved by Animal Care and Use Committee of Chiba Cancer Center Research Institute.

## Author Contributions

AT and AN conceived and designed the experiments. AT, SH, AO, JA, and YN performed the experiments. AT, SH, and AN analyzed the data. YN contributed reagents/materials/analysis tools. AT and AN wrote the paper. All authors contributed to the article and approved the submitted version.

## Funding 

This work was supported by a Grant-in-Aid from the Japan Ministry of Health, Labour and Welfare for Third Term Comprehensive Control Research for Cancer to AN, JSPS KAKENHI Grant Number JP21390317, JP24249061 to AN, JP19890276 to AT, MEXT KAKENHI Grant Number JP22791016 to AT, and a grant from the Takeda Science Foundation to AN.

## Conflict of Interest

The authors declare that the research was conducted in the absence of any commercial or financial relationships that could be construed as a potential conflict of interest.

## Abbreviations

ALK, anaplastic lymphoma kinase; DMEM, Dulbecco’s Modified Eagle Medium; BrdU, 5-bromo-2-deoxyuridine; DTSSP 3,3′-Dithiobis(sulfosuccinimidylpropionate); EGF, epidermal growth factor; EGFR, epidermal growth factor receptor; FBS, fetal bovine serum; FCM, flow cytometric analysis; FNIII, fibronectin type II; IHA, hemagglutinin; Hpfs, high power fields; Ig, immunoglobulin-like; IGF, insulin-like growth factor; LRR, leucine-rich repeats; N1mAb, monoclonal antibodies against the extracellular domains of NLRR1; NB, neuroblastoma; NLRR, neuronal leucine-rich repeat; mAbs, monoclonal antibodies; PBS, phosphate-buffered saline; qRT-PCR, quantitative real-time RT-PCR; RTK, Receptor tyrosine kinases.
