# Supplementary material for: NLRR1 Is a Potential Therapeutic Target in Neuroblastoma and MYCN-Driven Malignant Cancers
Source: Front Oncol. 2021 Jun 25;11:669667. doi: 10.3389/fonc.2021.669667 (PMC8279747; doi:10.3389/fonc.2021.669667)
Supplement: Supplementary file 1 [file DataSheet_1.pdf]

Figure S1

Takatori et al.

A

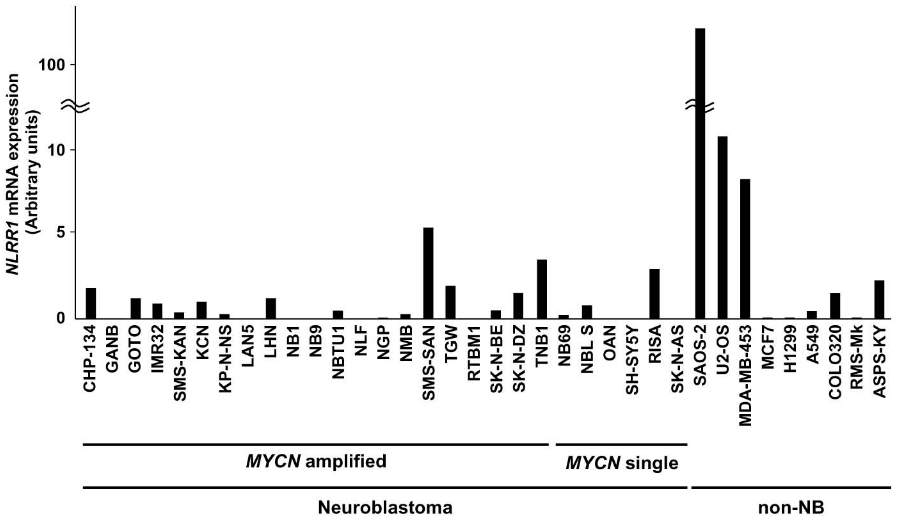

B

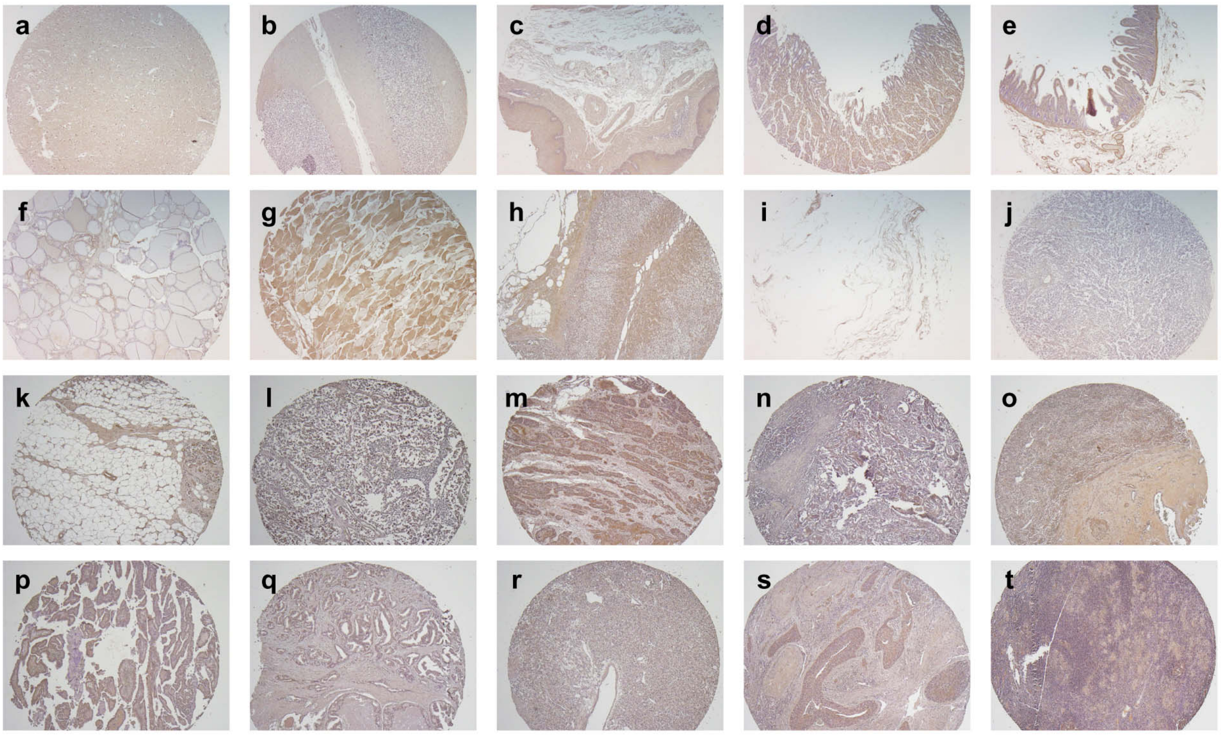

C

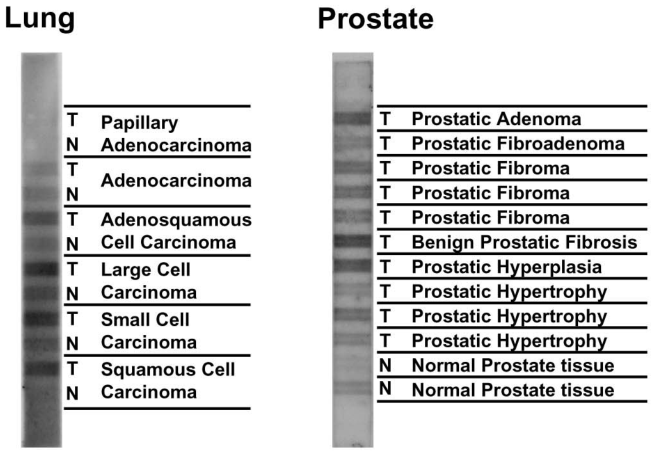

Figure S2

Takatori et al.

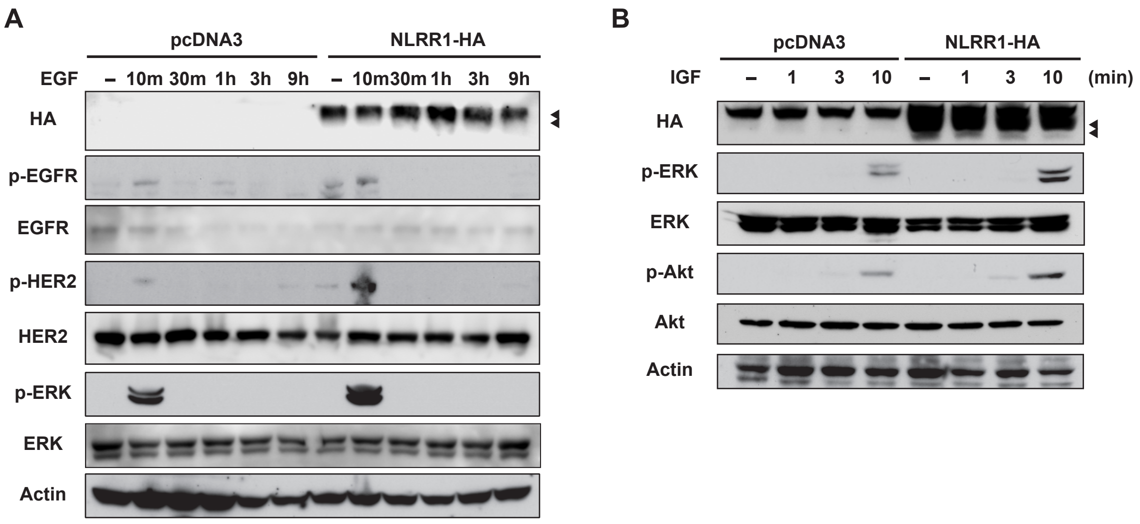

Figure S3

Takatori et al.

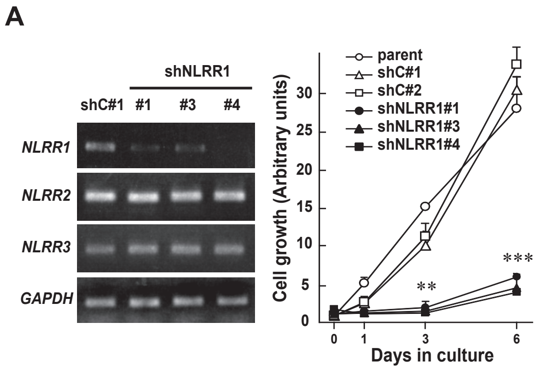

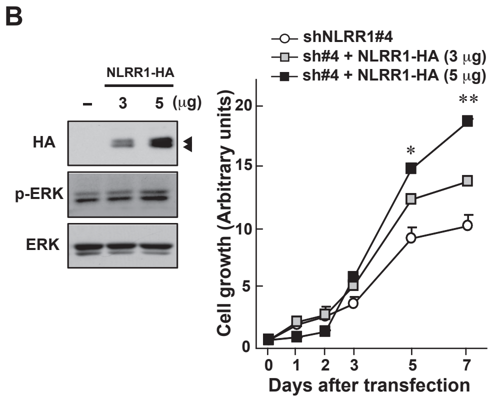

Figure S4

Takatori et al.

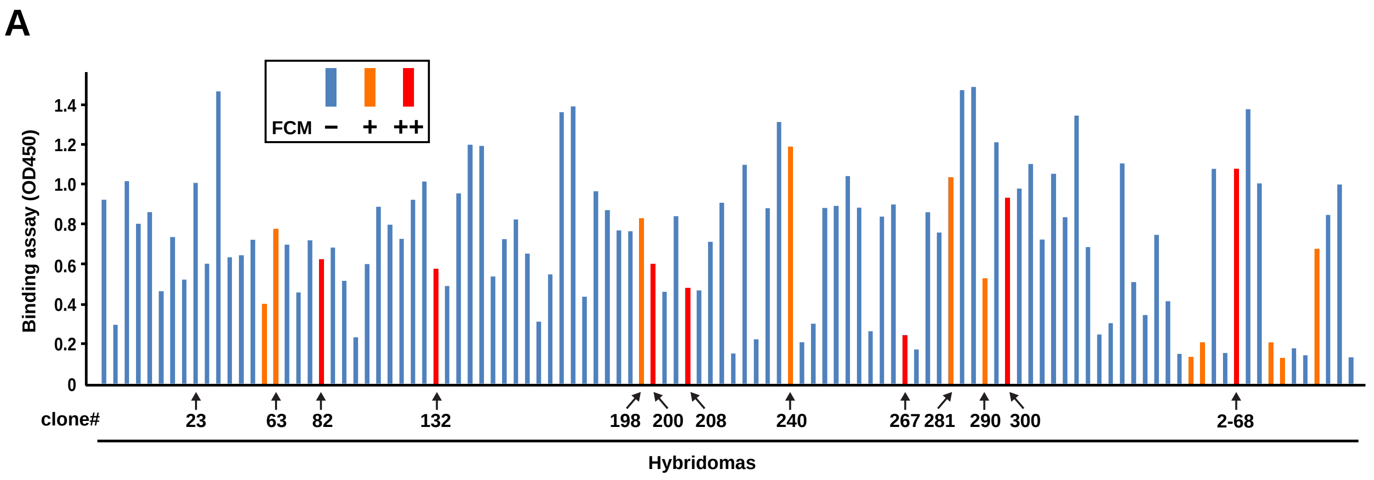

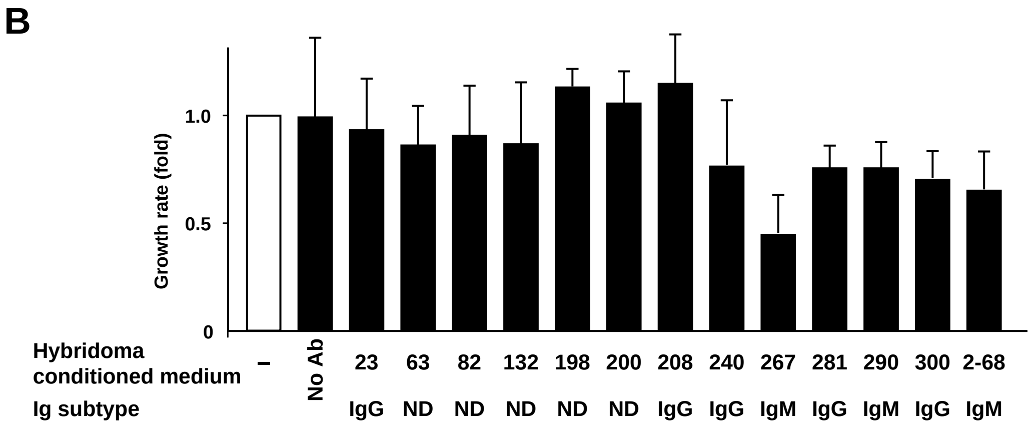

Figure S5

Takatori et al.

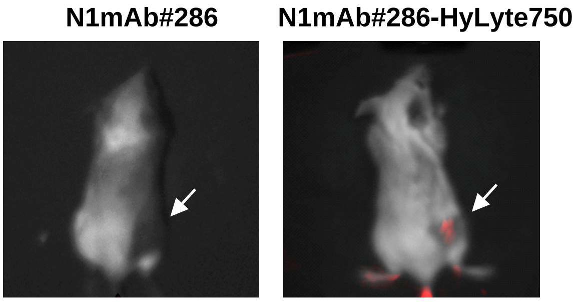

Figure S6

Takatori et al.

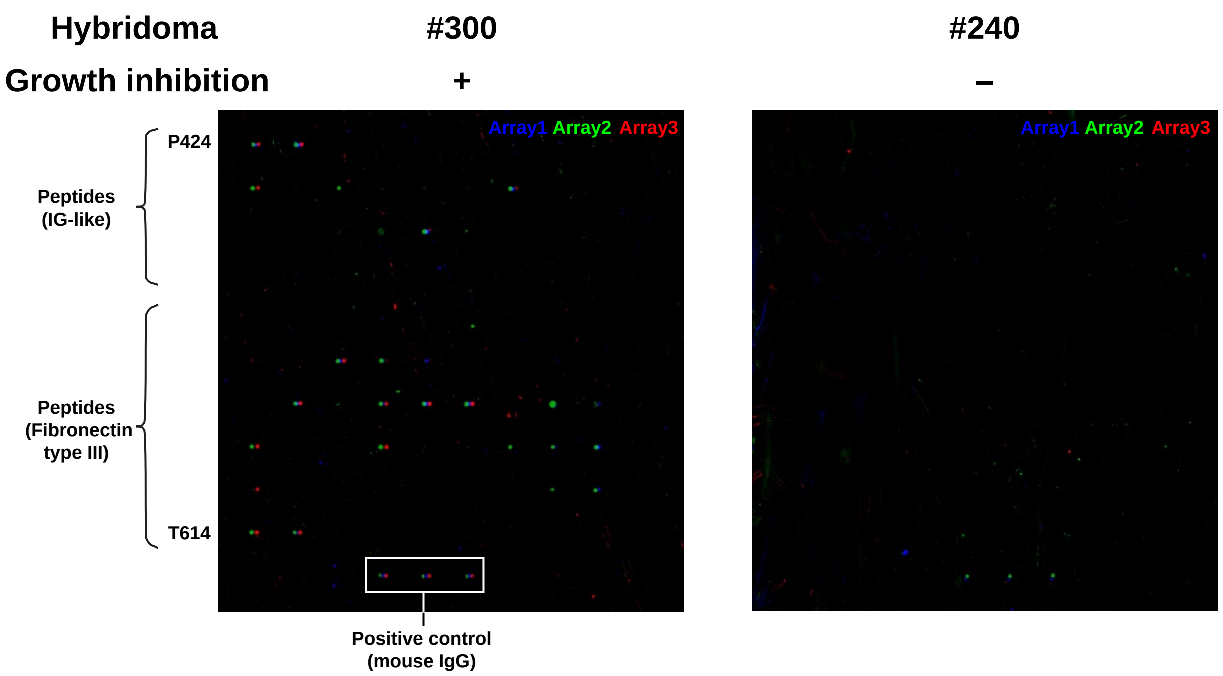

A

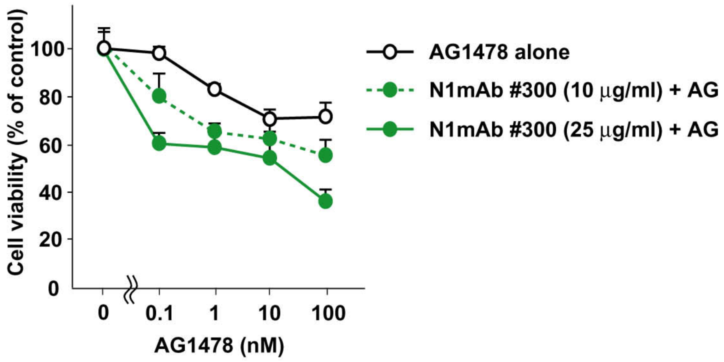
Cell viability (% of control)
100
80
60
40
20
0
0
0.1
1
10
100
AG1478 (nM)
AG1478 alone
N1mAb #300 (10 µg/ml) + AG
N1mAb #300 (25 µg/ml) + AG

B

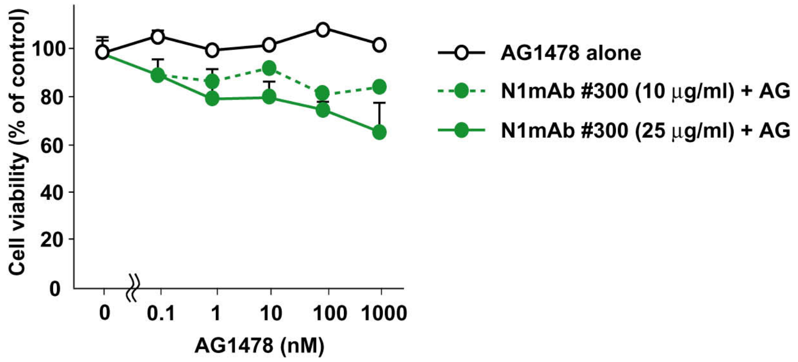
Cell viability (% of control)
100
80
60
40
20
0
0
0.1
1
10
100
1000
AG1478 (nM)
AG1478 alone
N1mAb #300 (10 µg/ml) + AG
N1mAb #300 (25 µg/ml) + AG

Figure S8

Takatori et al.

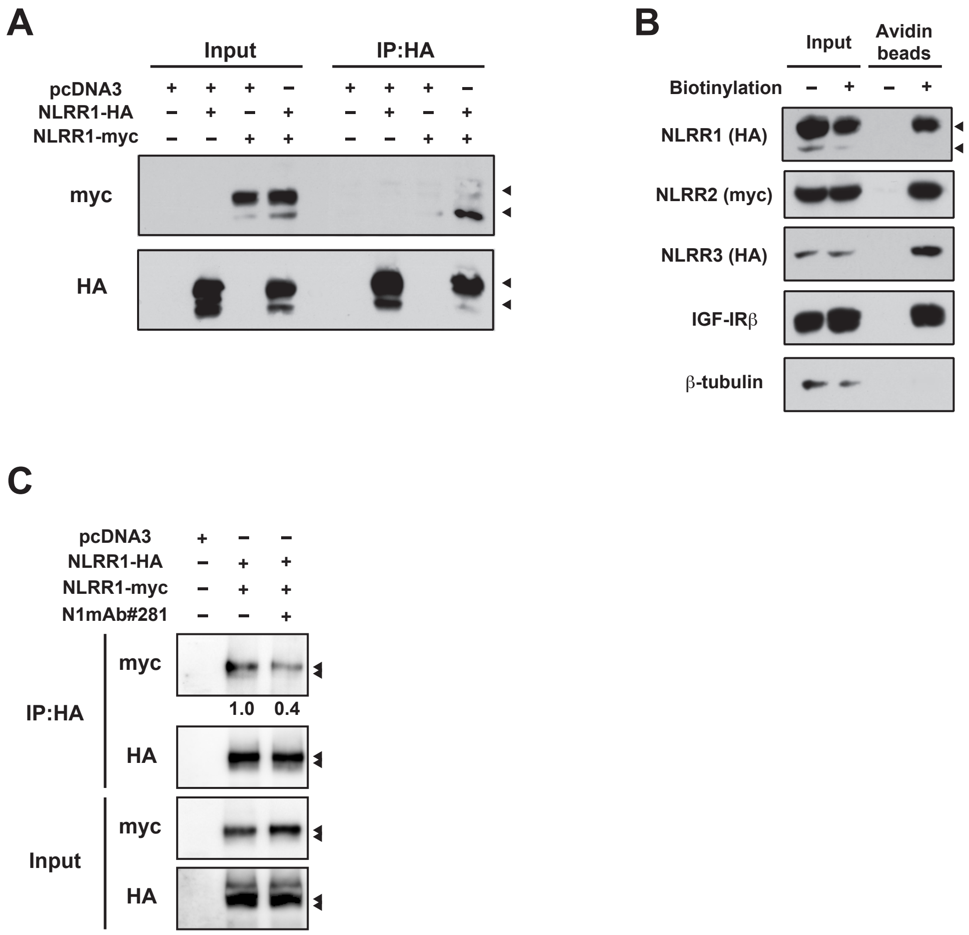

Supplementary Figure Legends

Figure S1. NLRR1 expression is upregulated in various types of cancers. (A) Expression of NLRR1 mRNA in NB and non-NB cell lines. Relative expression level of *NLRR1* mRNA was determined by calculating the ratio between *β-actin* and *NLRR1*. (B) Immunohistochemistry for NLRR1 in human normal and cancer tissues. Anti-N-terminal NLRR1 antibody was used to detect NLRR1 expression in tissue array sections of normal cerebrum (a), normal cerebellum (b), normal esophagus (c) normal stomach (d), normal small intestine (e), normal thyroid (f), normal skeletal muscle (g), normal adrenal gland (h), normal thymus (i), normal lymph node (j), breast infiltrating duct carcinoma (k), lung adenocarcinoma (l), esophagus squamous cell carcinoma (m), stomach adenocarcinoma (n), duodenum gastrointestinal stromal tumor (o), thyroid papillary carcinoma (p), prostate adenocarcinoma (q), kidney renal cell carcinoma (r), uterine cervix squamous cell carcinoma (s) and Hodgkin's lymphoma (t). Representative images are shown. (C) Tissue lysate arrays from lung and prostate normal and tumor tissues were immunostained with polyclonal anti-NLRR1 antibody.

Figure S2. Downstream signals upon EGF and IGF treatment are enhanced by NLRR1 expression. Enhanced ERK activation upon EGF and IGF treatment in a time-dependent manner. MCF7 cells were transfected with pcDNA3-NLRR1 and treated with 10 ng/ml EGF (A) or IGF (B). After treatment for the indicated time, cell lysates were collected and subjected to western blot analysis to detect the phosphorylation status of EGFR, HER2, ERK and Akt.

Figure S3. Knockdown of NLRR1 expression inhibits cell proliferation. (A) Knockdown of NLRR1 by introducing stable expression of shRNA against NLRR1 (shNLRR1) causes intensive growth inhibition in SK-N-BE compared with cells stably expressing control shRNA (shC). Expression levels of NLRR1 mRNA were checked by RT-PCR (left panel). Cells were seeded in 96-well plates at 500 cells per well and cell proliferation was examined by WST-8 assays (right panel). (B) Recovery of cell proliferation in NLRR1-stable knockdown SK-N-BE cells. Cells stably expressing NLRR1 shRNA #4 targeting the 3'-UTR of *NLRR1* were transiently transfected with

NLRR1 expression vector. The cells were seeded in 96-well plates at 2000 cells per well and subjected to WST-8 assays. Arrowheads, glycosylated NLRR1.

Figure S4. Screening for N1mAbs with growth inhibitory effect. (A) Cultured supernatants of the hybridomas producing antibodies against NLRR1 were analyzed by flow cytometric analysis (FCM) and the binding assay to NLRR1 proteins. (B) The culture supernatants positive for FCM and/or the binding assay were tested for growth inhibitory effect by culturing CHP134 cells at 1 × 10^5/ml in the medium containing 50% of the conditioned medium from the hybridomas.

Figure S5. *In vivo* fluorescence imaging with HiLyte Fluor 750-labeled N1mAb 281. Fluorescence images of xenograft tumor from NLRR1-expressing SH-SY5Y cells were acquired 24 h after intravenous injection of HiLyte Fluor 750-labeled N1mAb 281. The near-infrared fluorescent signal was detected from the tumor.

Figure S6. N1mAb 300 also detected Ig and FNIII domains of NLRR1. Microarrays of 91 overlapping 12-mer peptides from Ig and FNIII domains of NLRR1 were immunostained with N1mAbs 300 and 240. Bound antibodies were detected with Alexa 546-labeled anti-mouse IgG. The scanned arrays obtained from three independent experiments were indicated.

Figure S7. Treatment of N1mAb potentiates growth inhibitory effect of EGFR inhibitor. CHP134 (A) and A549 (B) cells were treated with N1mAb 300 and different concentrations of AG1478 (AG). Quantification of cell proliferation was performed by WST-8 assays. Data were normalized to the results for untreated cells and represented as percentage of control (mean ± SD).

Figure S8. NLRR1 proteins localize on cell surface and form self-multimer.

(A) HA-tagged and myc-tagged NLRR1 were expressed in HEK293 cells and the cell lysates were collected after crosslinking with membrane-impermeable DTSSP. Immunoprecipitation was performed using anti-HA antibody and the immunoprecipitates were subjected to western blot

analyses. Arrowheads, glycosylated NLRR1. (B) NLRR family proteins were overexpressed in HEK293 cells. Proteins on cell surface were biotinylated and the cell lysates were collected. Biotinylated proteins were isolated with avidin beads and subjected to western blot analyses. IGF-IR and β-III tubulin were used as positive and negative controls, respectively. (C) Co-immunoprecipitated myc-tagged NLRR1 with HA-tagged NLRR1 was reduced by N1mAb 281 treatment. HEK293 cells expressing HA-tagged and myc-tagged NLRR1 were treated with N1mAb 281 (25 μg/ml) for 3 h. The cell lysates were collected after crosslinking by DTSSP treatment and subjected to immunoprecipitation using anti-HA antibody.
